# Cognitive-Behavioural Social Skills Training: Mediation of Treatment Outcomes in a Randomized Controlled Trial for Youth at Risk of Psychosis: L’entraînement aux compétences sociales cognitivo-comportementales : variables médiatrices des résultats thérapeutiques dans le cadre d’un essai clinique randomisé pour les jeunes présentant un risque de psychose

**DOI:** 10.1177/07067437241295636

**Published:** 2024-11-11

**Authors:** Daniel J. Devoe, Lu Liu, Amy Braun, Kristin S. Cadenhead, Barbara A. Cornblatt, Eric Granholm, Jean Addington

**Affiliations:** 1 Department of Psychology, Mount Royal University, Calgary, Alberta, Canada; 2 Department of Psychiatry, Hotchkiss Brain Institute, University of Calgary, Calgary, Alberta, Canada; 3 Department of Psychiatry, 8784 University of California, San Diego, San Diego, California, USA; 4 Department of Psychiatry, Zucker Hillside Hospital, Long Island New York, USA

**Keywords:** psychosocial treatment, clinical high-risk, functioning, negative symptoms

## Abstract

**Objectives:**

Currently, there are no effective treatments for functional outcomes (i.e., role and social) and negative symptoms for youth at clinical high-risk (CHR) for psychosis. Investigations into possible mechanisms that may contribute to the improvement of functioning and negative symptoms are needed in CHR research to help inform psychosocial treatments. The present study examined whether functioning and negative symptoms were mediated by asocial beliefs, defeatist beliefs, self-efficacy, maladaptive schemas, anxiety, depression, social cognition, or attenuated psychotic symptoms (APS) in a large clinical trial.

**Methods:**

CHR participants (*n* = 203; 104 females; 99 males) were recruited as part of a three-site randomized control trial comparing group cognitive-behavioural social skills training (CBSST) versus a supportive therapy group. Mediation analyses were conducted to test the relationships between treatment group, mediators (asocial beliefs, defeatist beliefs, self-efficacy, maladaptive schemas, anxiety, depression, social cognition, and APS), and outcome (social and role functioning, and negative symptoms). The mediation analyses employed conditional process path analysis via ordinary least squares regression.

**Results:**

At the end of treatment, but not 12-month follow-up, more severe APS were found to mediate the impact of treatment on negative symptoms, and social and role functioning. The greater the severity of APS, the less likely that CBSST would result in improvement in negative symptoms and social and role functioning. Many of the other variables showed significant associations with social (less for role) functioning and negative symptoms but did not mediate the effect of treatment on these outcomes at the end of treatment or 12-month follow-up.

**Conclusions:**

There were no significant mediators except for APS at the end of treatment. Since more severe APS may result in participants being unable to fully participate in therapy and thus limit their gains, clinical implications may include offering some individual therapy to prepare these young people to benefit from the group treatment.

## Résumé

**Objectifs:**

actuellement, il n’existe pas de traitements efficaces pour améliorer les résultats fonctionnels (c.-à-d. scolaire/professionnel et social) et réduire les symptômes négatifs chez les jeunes présentant un risque clinique élevé de psychose. La recherche sur le risque clinique élevé doit se pencher sur les mécanismes susceptibles de contribuer à l’amélioration du fonctionnement et à la diminution des symptômes négatifs, afin d’orienter les traitements psychosociaux. La présente étude cherchait à déterminer si le fonctionnement et les symptômes négatifs sont influencés par les croyances asociales, les croyances défaitistes, la connaissance de ses propres capacités, les schémas inadaptés, l’anxiété, la dépression, la cognition sociale ou les symptômes psychotiques atténués (SPA) dans le cadre d’un vaste essai clinique.

**Méthodes:**

les participants présentant un risque clinique élevé (*n* = 203; 104 femmes; 99 hommes) ont été recrutés dans le cadre d’un ECR en trois groupes comparant l’entraînement en groupe aux compétences sociales cognitivo-comportementales (CBSST) à un groupe de thérapie de soutien. Des analyses de médiation ont été menées pour tester les relations entre le groupe étudié, les variables médiatrices (croyances asociales, croyances défaitistes, connaissance de ses propres capacités, schémas inadaptés, anxiété, dépression, cognition sociale et SPA) et les résultats (fonctionnement social et scolaire/professionnel, et symptômes négatifs). Les analyses de médiation ont fait appel à l’analyse des processus conditionnels par régression ordinaire des moindres carrés ordinaires.

**Résultats:**

à la fin du traitement, mais pas lors du suivi à 12 mois, on a constaté que la présence de SPA plus sévères médiait les effets du traitement sur les symptômes négatifs, ainsi que sur le fonctionnement social et scolaire/professionnel. Plus les SPA sont graves, moins le CBSST est susceptible d’entraîner une diminution des symptômes négatifs et une amélioration du fonctionnement social et scolaire/professionnel. De nombreuses autres variables ont révélé des associations significatives avec le fonctionnement social (moins avec le fonctionnement scolaire/professionnel) et les symptômes négatifs, mais n’ont pas influencé l’effet du traitement sur ces résultats à la fin du traitement ou lors du suivi à 12 mois.

**Conclusions:**

il n’y a pas de variables médiatrices significatives à l’exception des SPA à la fin du traitement. Étant donné que des SPA plus graves peuvent empêcher les participants de participer pleinement à la thérapie et donc limiter les bénéfices qu’ils pourraient en retirer, il serait bon, d’un point de vue clinique, de proposer une thérapie individuelle afin de préparer ces jeunes à tirer profit de la thérapie de groupe.

## Introduction

Over the past two decades, a considerable amount of research has focused on youth at clinical high-risk (CHR) of developing psychosis, with the main objective being the prevention of transitioning to a psychotic disorder.^1^ However, most CHR youth will not develop a psychotic disorder.^2^ CHR youth who do not transition to a psychotic disorder are often characterized by having functional deficits (social and role) which significantly impact their day-to-day lives.^3^ Furthermore, CHR youth often exhibit persistent negative symptoms, which can be predictive of a transition to a psychotic disorder.^4–8^ Furthermore, a consistent relationship has been demonstrated between social and role functioning and negative symptoms in CHR youth.^9^

A meta-analysis of 19 studies examining functioning demonstrated that overall no treatments significantly impacted functioning and that none of these treatment trials utilized functioning as a primary outcome.^10^ Since then several other trials have explored the impact of treatment on functioning in CHR youth. One small pilot trial found significant improvements in social functioning when treated with integrated social and cognitive remediation therapy compared to active control.^11^ Another trial found improvement in functioning when treatment as usual was coupled with acceptance and commitment therapy.^12^ However, one study found that cognitive remediation therapy compared to treatment as usual had no impact on social functioning.^13^ Finally, an Omega-3 trial found improvement for functioning at 6- and 12-month follow-up compared to placebo.^14^ In terms of negative symptoms, a meta-analysis demonstrated that very few studies examined negative symptoms (*n* = 11) and that overall no treatments significantly impacted negative symptoms in CHR youth.^15^ Thus, investigations into possible mechanisms that may contribute to the improvement of functioning and negative symptoms are needed in CHR youth to help inform psychosocial treatments.

Potential mediators of treatment for negative symptoms and functioning in CHR youth may include attenuated psychotic symptoms (APS), asocial beliefs, defeatist beliefs, self-efficacy, maladaptive schemas, social cognition, anxiety, and depression. Beck et al.^16,17^ proposed that defeatist beliefs (e.g., “If I fail at my school, then I am a failure as a person”) and asocial beliefs (e.g., “Making new friends isn’t worth the energy it takes”) contribute to negative symptoms and poor functioning in schizophrenia. CHR youth have substantial asocial beliefs and defeatist beliefs and increased asocial and defeatist beliefs have been associated with poorer negative symptoms in CHR youth.^18,19^ Additionally, increased defeatist beliefs have been associated with poorer role functioning and increased asocial beliefs with poorer social functioning.^18^

Furthermore, CHR youth have lower social self-efficacy (e.g., confidence in going to a party with friends) compared to healthy controls.^20,21^ Poorer social self-efficacy has been associated with more severe negative symptoms and poorer social functioning in CHR youth.^18^ In terms of maladaptive schemas, increased negative-self schemas have been reported in a CHR sample with persistent negative symptoms versus those without,^22^ and more severe negative symptoms have been associated with negative-self schemas.^18^

For social cognition, one study demonstrated that the combination of facial affect processing and negative symptoms was the best model for predicting transition to psychosis,^23^ while other studies have demonstrated that social cognition is associated with functional deficits in CHR youth^24^ and negative symptoms.^25^ In contrast, other studies have not shown a relationship between social cognition and negative symptoms.^26,27^

Studies examining depression have demonstrated a link between both negative symptoms and functioning in CHR youth.^28,29^ One study found that CHR youth with major depressive disorder (MDD) had increased negative symptoms and lower social and role functioning compared to CHR without MDD.^28^ In another study, CHR youth with either current or past depression presented with significantly more severe negative symptoms, and greater deficits in social functioning.^29^ Likewise, social anxiety has been negatively associated with social functioning in CHR youth,^30^ with sustained social anxiety linked to poorer long-term functional outcomes,^31^ and more severe social anxiety with more severe negative symptoms.^32,33^ Finally, when examining APS in CHR youth, APS total scores have been observed to be a significant predictor of poorer social functioning.^34^

The current study examines data from a three-site randomized control trial (RCT) comparing the effects of cognitive-behavioral social skills training (CBSST) with a supportive therapy (ST) in CHR youth.^35^ CBSST is a group intervention which combines elements of cognitive-behaviour therapy (CBT) and social skills training (SST). The CBSST manual was adapted to reflect the ages and APS of CHR youth.^36^ The primary aim of this RCT was to examine the impact of treatment on social and role functioning. High-fidelity CBT and SST interventions were delivered in CBSST with comparable high fidelity across the three sites. More details on fidelity and outcomes can be found in Addington et al. and Kelsven et al.^37,38^ As reported in the original outcome paper, there were no significant differences between the CBSST and ST groups at the end of treatment nor at the 12-month follow-up.^37^ Thus, the next step is to examine mediators of treatment for social and role functioning as well as negative symptoms which may provide insights into the development of future treatments.

The primary aim of this current study was to explore APS, asocial beliefs, defeatist beliefs, self-efficacy, maladaptive schemas, anxiety, depression, and social cognition as potential mediators of treatment of negative symptoms and functioning.

## Materials and Methods

### Setting and Participants

All CHR participants (*N* = 203) were recruited in a three-site (University of Calgary, University of California at San Diego, and Zucker-Hillside Hospital) RCT: Recovery through Group (ReGroup). More study details are available in the original methods and outcomes papers.^35,37^ For the current study, the final sample for the mediation analyses included 152 CHR participants (CBSST = 70, ST = 82).

CHR participants were referred to this trial by several external sources (i.e., healthcare providers, educators, social services) or were self-referred. Telephone screening was utilized to exclude any youth who may already be psychotic. Participants that could potentially meet the Criteria of Psychosis-risk Syndromes (COPS)^39^ were invited to an in-person eligibility evaluation and consent.

CHR participants were included in the trial if they met the following criteria: (1) between 13 and 30 years old; (2) could comprehend and sign an informed consent form (or assent for minors) in English; (3) presently meet or have met criteria for a psychosis-risk syndrome as per COPS criteria in the past 4 years; (4) at least one positive symptom on the Scale of Psychosis-risk Symptoms (SOPS) symptom rated 3 (moderate) and no symptom rated 6 (severe or psychotic); (5) ratings on either the Global Functioning: Social Scale or Global Functioning: Role Scale were rated 7 or less (7 = mild problems/impairment).

Exclusion criteria included: (1) previously met criteria for a lifetime Axis 1 psychotic disorder; (2) had an IQ < 70; (3) past or current history of a central nervous system disorder; (4) any substance dependence in the past 3 months; and (5) the diagnostic psychosis-risk symptoms are or were clearly caused by an Axis 1 disorder.

### Interventions

In this trial, one group received CBSST while the other received ST. CBSST is a manualized 18-week group treatment consisting of three modules: (1) cognitive skills, (2) social skills, and (3) problem-solving skills. Each CBSST module includes a weekly group session over 6 weeks. Several studies have examined the efficacy of CBSST in people with schizophrenia,^40–42^ and a practical published treatment manual describes all modules in detail.^43^ The CBSST manual was adapted to reflect the ages and attenuated symptoms of CHR youth. These changes and treatment details with case studies are outlined in detail in Kelsven et al.^36^ Content for each of the six sessions in each of the three modules plus details of manual modifications for CHR youth is presented in the Supplementary material.

ST served as the control treatment in this trial. Participants in this group received 18 weeks of group therapy aimed at controlling for therapist and peer group contact, social interaction, and support. Individuals in the ST group did not receive instruction in CBSST techniques. Instead, educational materials regarding CHR for psychosis, and comorbid disorders were provided. In the ST group, the therapists focused on listening, reflecting, understanding, and demonstrating acceptance.

### Assessments

#### CHR Criteria

Participants were assessed for being at CHR for psychosis using the COPS, based on the Structured Interview for Psychosis-risk Syndromes (SIPS).^39^

#### Mediator Variables

APS was evaluated using the SOPS positive symptom subscale.^39^ This includes unusual thought content (p1), suspiciousness/persecutory ideas (p2), grandiose ideas (p3), perceptual abnormalities (p4), and disorganized communication (p5) for a maximum total score of 30.

The Defeatist Performance Attitude Scale (DPAS) was used to assess defeatist performance attitudes. The DPAS is a 15-item self-report that rates defeatist attitudes on a 1–7 Likert scale, higher scores signify more severe defeatist beliefs (range = 15–105).^44^

Asocial Beliefs were measured with the 15-item Asocial Beliefs Scale (range = 0–15).^45,46^ Items are rated true or false, with higher scores demonstrating more severe asocial beliefs.

The Social Self-Efficacy subscale (SSES) from the Revised Self-Efficacy Scale was utilized to assess social self-efficacy.^47^ This 19-item scale (rated 0%–100%) measures how confident participants are in performing everyday social behaviours. Higher scores demonstrate more self-efficacy.

The Brief Core Schema Scale (BCSS) is a self-report scale used to assess negative and positive schemas.^48,49^ Only BCSS negative-self and negative-others scores were utilized. Higher scores indicate more maladaptive schemas.

To measure anxiety the Social Interaction Anxiety Scale (SIAS),^50^ and the Social Anxiety Scale (SAS)^51^ were used. The SIAS, a 20-item scale, measures anxiety around social interactions on a scale from 0 to 4 (0 = not at all characteristic or true of me, 4 = extremely characteristic or true of me). The SAS, a 20-item scale, measures generalized anxiety on a 4-point scale (1 = a little of the time, 4 = most of the time).

Depression was assessed with the Calgary Depression Scale for Schizophrenia (CDSS),^38^ which has been validated in CHR samples.^52^ The CDSS is clinician rated on nine items that assess levels of depression in patients for a total possible score of 27.

The Wechsler Abbreviated Scale of Intelligence (WASI-2) vocabulary and matrix reasoning tasks^53^ were used to assess participants’ intellectual capacity.

Social Cognition was assessed with two measures of facial affect and a measure of Theory of Mind (ToM). For facial affect the Penn Emotion Recognition (ER40) and the Penn Emotion Differentiation (EDF40) tasks were used.^54,55^ For ToM, the Social Inference subscale of The Awareness of Social Inference Test (TASIT) was utilized.^56^

#### Outcome Variables

Assessment of functioning was measured utilizing the Global Functioning: Social (GF:S) and the Global Functioning: Role (GF:R) scales.^57,58^ The GF:S measures the level of social contact, amount and quality of friendships, intimate relationships, and connection with family members. The GF:R measures the level of functioning at school or employment. The GF:S and GF:R are both rated on a 10-point scale. Higher scores indicate better functioning.

Negative symptoms were evaluated using the SOPS negative symptom subscale.^39^ For the analysis only the SOPS negative symptoms scale scores of social anhedonia (N1), avolition (N2), expression of emotion (N3), experience of emotions and self (N4), and ideational richness (N5) were utilized for a maximum total score of 30. Occupational functioning (N6) was not utilized as it overlaps with the GF:R and GF:S.

### Procedures

Raters were experienced research clinicians who demonstrated excellent reliability compared to “gold standard” ratings on the SOPS (ICC = 0.91), GF:S (ICC = 0.89) and GF:R and GF:S (ICC = 0.89). This study was approved by the Institutional Review Boards of all three sites. Written informed consent, including parental consent, was obtained from all adult participants and parents/guardians of minors. A Data and Safety Monitoring Board (DSMB) was set up with National Institute of Mental Health (NIMH) approved members. Calls with the DSMB and site principal investigators occurred every 4 months. All therapy groups were in-person.

### Analyses

Three mediation models were analyzed using Hayes Process Macro Model 4,^59^ in SPSS a powerful tool for conducting conditional process path analysis employing ordinary least squares regression. The first and second mediation models examined the “*a* path” from group treatment (coded as 0 and 1) to all mediator variables, the “*b* path” from all mediator variables to social and role functioning and mediated effects (*ab*) of group treatment on social and role functioning through all mediator variables. The third model explored the effect of group treatment on negative symptoms through mediator variables, respectively. This PROCESS model utilized bias-corrected bootstrap confidence intervals (CIs) for inference about indirect effects. It was executed with standardized effects, showing total effect models, enabling computation of the 95% CI for the indirect effect (path *a* × path *b*). A total of 5,000 bias-corrected bootstrap samples were employed for all PROCESS tests.^59,60^

## Results

### Sample Characteristics

**Figure 1.**
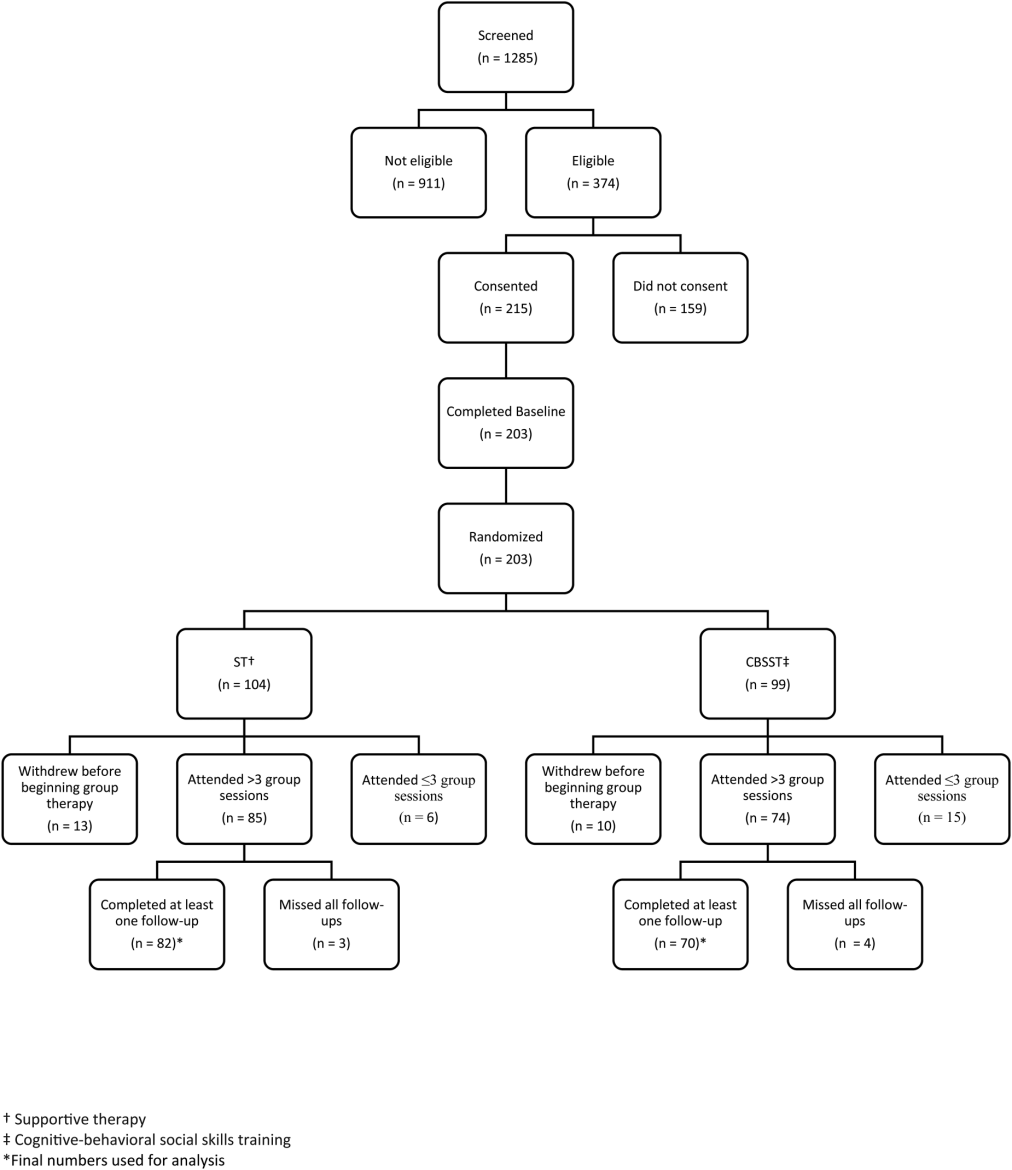
Consort diagram.

Details of recruitment and follow-up are presented in Figure 1, the Consort Diagram, from the first outcome paper.^37^ Of the 215 participants who consented, 203 completed the baseline assessment and were subsequently randomized. Of the 203 randomized, 23 withdrew before beginning therapy, 21 attended three or less sessions, and seven participants did attend several group sessions (range 5–15) but were unavailable for any follow-up assessments. These 51 participants were not included in the final analyses (CBSST = 29, ST = 22). The final sample for analyses included 152 participants (CBSST = 70, ST = 82).

Those who were excluded (*N* = 51) were compared to those included (*N* = 152). As reported in the first outcome paper,^37^ the groups did not differ significantly on any variables (see Supplementary Table 1).

There were no differences in any demographics between the treatment groups (see Table 1). There were no differences in the mean number of group sessions attended by each group (mean (SD) for CBSST = 13.8 (3.5), and for ST = 13.8 (3.3).

**Table 1.** Demographic Comparisons at Baseline.

Variable	CBSST *n *= 70	ST *n *= 82	Test statistic	Significance value
	Mean (SD)	Mean (SD)	*t*	*P*
Age (years)	17.36 (4.01)	17.49 (4.12)	−0.20	0.844
Years of education	10.30 (2.68)	10.45 (2.64)	−0.35	0.727
IQ	103.00 (12.24)	104.00 (14.90)	−0.48	0.631
	Frequency (%)	Frequency (%)	χ^2^	*P*
Sex				
Male	29 (41.4)	40 (48.8)	0.82	0.364
Female	41 (58.6)	42 (51.2)		
Race				
Caucasian	41 (58.6)	51 (62.2)	0.22	0.895
Black	8 (11.4)	9 (11.0)		
Other ^a^	21 (30.0)	22 (26.8)		
Marital status				
Single/never married	67 (95.7)	79 (96.3)	0.04	0.843
Married/common law	3 (4.3)	3 (3.7)		
Living arrangement				
Living with family/spouse	66 (94.3)	76 (92.7)	0.16	0.691
Independent ^b^	4 (5.7)	6 (7.3)		

^a^ Includes First Nations, East Asian, Southeast Asian, South Asian, West/Central Asian, and Middle Eastern, Native Hawaiian or Pacific Islander, and mixed race.

b Includes living with friends (excluding spouse/partners), in a boarding/group home, academic residence, or living alone.

Mean values for all variables over time are presented in Supplementary Table 2, previously reported in Addington et al.^37^

### Mediation Analyses

Results of models estimating the “*a* path” [treatment to mediator], “*b* path” (mediator to outcome) and mediated effects (*ab*) of treatment on social functioning, role functioning and negative symptoms through all mediator variables are shown in Tables 2–4 for end of treatment and 12-month follow-up.

**Table 2.** Mediated Effects (*ab*) of Treatment Group (CBSST vs. ST) on Global Functioning: Social Through Mediators at End of Treatment and 12-Month Follow-up.

End of treatment					
Mediator	Effect of IV on mediator (*a* path)	Effect of mediator (*b* path)	Indirect effect (*ab* path)	Lower CI	Upper CI
SOPS-positive	−1.6006*	−0.1080***	0.1729	0.0163	0.4315
CDSS	−0.3672	−0.0984	0.0361	−0.0947	0.1814
SIAS	−1.7712	−0.0202***	0.0357	−0.1095	0.1817
SAS	−0.5312	−0.0153	0.0081	−0.0711	0.0834
BCSS-self	−1.1169	−0.0521**	0.0581	−0.0549	0.1948
BCSS-other	0.7183	−0.0749***	−0.0538	−0.2343	0.0981
DPAS	−3.3294	−0.0223***	0.0741	−0.0604	0.2105
ABS	−0.0342	−0.1489***	0.0051	−0.1732	0.1906
SSES	3.5076	0.0237***	0.0833	−0.1176	0.2789
ER40_CR	0.8548	−0.0027	−0.0023	−0.0857	0.0659
EDF40ED	−1.7841	0.0028	−0.0050	−0.0878	0.0802
TASIT	−0.3150	0.0239	−0.0075	−0.0753	0.0603
12-month follow-up					
SOPS-positive	−1.0486	−0.1217***	0.1276	−0.0412	0.3899
CDSS	−0.0021	−0.0932*	0.0002	−0.1079	0.1427
SIAS	−5.6842	−0.0207**	0.1179	−0.0126	0.3090
SAS	−1.9896	−0.0103	0.0205	−0.0400	0.1138
BCSS-self	−1.5533	−0.0488*	0.0758	−0.0137	0.2556
BCSS-other	−2.0208	−0.0460*	0.0929	−0.0050	0.2427
DPAS	−3.7165	−0.0202**	0.0751	−0.0674	0.2567
ABS	−0.0538	−0.1601***	0.0086	−0.2029	0.2278
SSES	4.0541	0.0296***	0.1201	−0.1205	0.3919
ER40_CR	0.6079	0.0260	0.0158	−0.0405	0.1031
EDF40ED	−1.1907	−0.0134	0.0160	−0.0476	0.1195
TASIT	−0.3469	0.0559*	−0.0194	−0.1618	0.1210

*Note.* SOPS-positive = Scale of Psychosis-Risk Symptoms (positive symptoms); CDSS = Calgary Depression Scale for Schizophrenia; SIAS = Social Interaction Anxiety Scale; SAS = Social Anxiety Scale Total Score; BCSS-self = Brief Core Schema Scale–negative self; BCSS-others = Brief Core Schema Scale–negative others; DPAS = Defeatist Performance Attitude Scale; ABS = Asocial Beliefs Scale; SSES = Social Self Efficacy Scale; ER40_CR = Facial Affect Recognition; EDF40ED = Facial Affect Discrimination; TASIT = The Awareness of Social Interference Test.

*P < 0.05, **P < 0.01, ***P < .001.

**Table 3.** Mediated Effects (*ab*) of Treatment Group (CBSST vs. ST) on Global Functioning: Role Through Mediators at End of Treatment and 12-Month Follow-up.

End of treatment					
Mediator	Effect of IV on mediator (*a* path)	Effect of mediator (*b* path)	Indirect effect (*ab* path)	Lower CI	Upper CI
SOPS-positive	−1.6006*	−0.1063*	0.1701	0.0091	0.4286
CDSS	−0.3672	−0.0831	0.0305	−0.0878	0.2065
SIAS	−1.7712	−0.0093	0.0164	−0.0726	0.1331
SAS	−0.5312	−0.0158	0.0084	−0.0893	0.0915
BCSS-self	−1.1169	−0.0318	0.0355	−1.0474	0.2057
BCSS-other	0.7183	−0.0486	−0.0349	−0.2025	0.0666
DPAS	−3.3294	−0.0040	0.0132	−0.0921	0.1194
ABS	−0.0342	−0.0472	0.0016	−0.0963	0.1032
SSES	3.5076	0.0081	0.0285	−0.0619	0.1565
ER40_CR	0.8548	0.0169	0.0144	−0.1182	0.1134
EDF40ED	−1.7841	0.0117	−0.0209	−0.1774	0.0938
TASIT	−0.3150	0.0388	−0.0122	−0.1562	0.0745
12-month follow-up					
SOPS-positive	−1.0486	−0.1867***	0.1958	−0.0717	0.5281
CDSS	−0.0021	−0.1272*	0.0003	−0.1888	0.1684
SIAS	−1.0123	−0.0307	0.0702	−0.0448	0.2844
SAS	−1.9896	−0.0087	0.0173	−0.0456	0.1225
BCSS-self	−1.5533	−0.0521	0.0809	−0.0266	0.3282
BCSS-other	−2.0208	0.0075	−0.0152	−0.1543	0.1142
DPAS	−3.7165	0.0030	−0.0110	−0.1220	0.0843
ABS	−0.0538	−0.0104	0.0006	−0.0841	0.0741
SSES	4.0541	0.0187*	0.0757	−0.0749	0.3218
ER40_CR	0.6079	0.0724	0.0440	−0.0462	0.2747
EDF40ED	−1.1907	0.0084	−0.0100	−0.1460	0.1083
TASIT	−0.3469	0.0746*	−0.0259	−0.2129	0.1803

*Note.* SOPS-positive = Scale of Psychosis-Risk Symptoms (positive symptoms); CDSS = Calgary Depression Scale for Schizophrenia; SIAS = Social Interaction Anxiety Scale; SAS = Social Anxiety Scale Total Score; BCSS-self = Brief Core Schema Scale–negative self; BCSS-others = Brief Core Schema Scale–negative others; DPAS = Defeatist Performance Attitude Scale; ABS = Asocial Beliefs Scale; SSES = Social Self Efficacy Scale; ER40_CR = Facial Affect Recognition; EDF40ED = Facial Affect Discrimination; TASIT = The Awareness of Social Interference Test.

*P < 0.05, ***P < .001.

**Table 4.** Mediated Effects (*ab*) of Treatment Group (CBSST vs. ST) on Negative Symptoms Through Mediators at End of Treatment and 12-Month Follow-up.

End of treatment					
Mediator	Effect of IV on mediator (*a* path)	Effect of mediator (*b* path)	Indirect effect (*ab* path)	Lower CI	Upper CI
SOPS-positive	−1.4134	0.3296***	−0.4658	−1.1454	−.0010
CDSS	−0.4133	0.4076***	−0.1685	−0.8484	0.3541
SIAS	−1.9502	0.0855***	−0.1667	−0.7974	0.3975
SAS	−0.2588	0.0563	−0.0146	−0.2853	0.3101
BCSS-self	−1.1433	0.1577**	−0.1803	−0.6942	0.1448
BCSS-other	0.6424	0.3406***	0.2188	−0.4517	0.9455
DPAS	−3.7118	0.0775***	−0.2877	−0.8737	0.1748
ABS	−0.0349	0.5547***	−0.0194	−0.7339	0.6145
SSES	3.6005	−0.0864***	−0.3110	−1.0977	0.3688
ER40_CR	0.7521	0.0684	0.0514	−0.1416	0.3057
EDF40ED	−1.6002	−0.1098	0.1757	−0.0735	0.5757
TASIT	−0.4946	−0.1833**	0.0907	−0.3227	0.5521
12-month follow-up					
SOPS-positive	−1.1037	0.5895***	−0.6507	−1.7757	0.1814
CDSS	−0.0646	0.5470***	−0.0353	−0.8079	0.5639
SIAS	−6.1242	0.1031***	−0.6316	−1.4274	0.0166
SAS	−2.1277	0.0783*	−0.1666	−0.5981	0.1821
BCSS-self	−1.6429	0.2661***	−0.4371	−1.2090	0.0407
BCSS-other	−2.1266	0.1671*	−0.3554	−0.9143	0.0072
DPAS	−4.3170	0.0678**	−0.2927	−0.8907	0.1656
ABS	−0.1461	0.5568***	−0.0813	−0.8148	0.6479
SSES	4.7544	−0.1276***	−0.6068	−1.7280	0.4533
ER40_CR	0.6420	−0.0932	−0.0599	−0.3464	0.2130
EDF40ED	−1.3036	−0.0728	0.0949	−0.1415	0.4824
TASIT	−0.3205	−0.2403**	0.0770	−0.5012	0.6854

*Note.* SOPS-positive = Scale of Psychosis-Risk Symptoms (positive symptoms); CDSS = Calgary Depression Scale for Schizophrenia; SIAS = Social Interaction Anxiety Scale; SAS = Social Anxiety Scale Total Score; BCSS = self, Brief Core Schema Scale–negative self; BCSS = others, Brief Core Schema Scale–negative others; DPAS = Defeatist Performance Attitude Scale; ABS = Asocial Beliefs Scale; SSES = Social Self Efficacy Scale; ER40_CR = Facial Affect Recognition; EDF40ED = Facial Affect Discrimination; TASIT = The Awareness of Social Interference Test.

*P < 0.05, **P < 0.01, ***P < .001.

#### Mediator Relation with Outcome Variables

The “*b* path” analyses, examining associations between all mediator variables and outcomes (social functioning, role functioning, negative symptoms), revealed several significant associations (Tables 2–4). At the end of treatment, (i) SOPS positive score, SIAS, BCSS negative-self and negative-others, DPAS, ABS, and SSES were associated with social functioning; (ii) SOPS positive score, was associated with role functioning; and (iii) SOPS positive score, CDSS, SIAS, BCSS negative-self and negative-others, DPAS, ABS, SSES, and TASIT were associated with negative symptoms. At 12-month follow-up, (i) SOPS positive score, CDSS, SIAS, BCSS negative-self and negative-others, DPAS, ABS, SSES, and TASIT were associated with social functioning; (ii) the SOPS positive total score, CDSS, SSES, and TASIT were associated with role functioning; and (iii) SOPS positive score, CDSS, SIAS, SAS, BCSS negative-self and negative-others, DPAS, ABS, SSES, and TASIT were associated with negative symptoms.

#### Estimation of Mediated Effects

Mediation effects (*ab* path) and associated 95% CIs were estimated. At the end of treatment, the SOPS-positive symptom score mediated the effects of treatment on social functioning, role functioning, and negative symptoms. No other mediator variables mediated the effect of treatment on social functioning, role functioning, or negative symptoms.

For 12-month follow-up, none of the mediator variables tested mediated the relationship between treatment group and social functioning, role functioning, or negative symptoms.

## Discussion

This study found that APS mediated the treatment effects on the outcomes of social functioning, role functioning, and negative symptoms at the end of treatment but not 12-month follow-up. In addition, several significant associations were found when examining the *b* path analyses on the outcomes of social functioning, role functioning, and negative symptoms at both end of treatment and 12-month follow-up.

For the mediation analyses, the goal was to better understand the pathway between treatment to social functioning, role functioning, and negative symptoms by exploring how APS, asocial beliefs, defeatist beliefs, social self-efficacy, maladaptive schemas, anxiety, depression, and social cognition might mediate these relationships (i.e., *ab* path). However, the treatment group effect was not mediated by any of these variables for the outcomes of social functioning, role functioning, and negative symptoms at any time point. The exception was that APS mediated the effects of treatment at the end of treatment. The greater the severity of APS, the less likely that CBSST would result in improvement in negative symptoms and social and role functioning. The current results are in contrast to a previous study in people with schizophrenia that found that the effect of treatment group (CBSST) on functioning and experiential negative symptoms was mediated through defeatist beliefs whereas asocial beliefs only mediated effects on experiential negative symptoms.^61^ The differences between the current and aforementioned study may be due to differences in methodology, sample populations, and study designs. One explanation of this discrepancy may be that in the schizophrenia study only experiential negative symptoms were mediated by defeatist beliefs and asocial beliefs,^61^ whereas in the current study, we examined both expressive and experiential negative symptoms together. Thus, a more granular approach to examining mediation of treatment via defeatist beliefs and asocial beliefs for experiential negative symptoms in CHR youth could be examined in future studies.

However, APS did significantly mediate the impact of both treatments (CBSST and ST) on social functioning, role functioning, and negative symptoms at the end of treatment (*ab* path). Moreover, APS was significantly associated with social and role functioning, and negative symptoms (*b* path) at the end of treatment. To the best of our knowledge, this is the first study to explore the mediated effects of APS on treatment outcomes in CHR. It is likely that more severe APS has an impact on behaviour or in this case participants’ ability to engage in CBSST. Since CBSST often relies on homework to help participants practice their skills, it is possible that the more APS they are experiencing, the less likely they are to be able to participate fully in the CBSST either in the sessions or with their homework and make the best use of it. This may in turn have contributed to poorer social and role functioning and increased negative symptoms. This aligns with previous research that has demonstrated that higher APS total scores have been observed to be a significant predictor of poorer social functioning.^34^ In addition, Tran et al., reported that the first 6 months of CHR program enrollment may be a critical window for improving negative symptoms with less improvement afterwards. This is one possible explanation for why there were no significant results between treatments and APS at 12-month follow-up for negative symptoms in the current analysis.^62^ Therefore, the first 6 months may represent a window of opportunity for early psychosocial interventions to target APS which may help improve social functioning, role functioning, and negative symptoms earlier in their course.

The strength of this study is that it was a well-conducted trial.^37^ There are, however, several limitations, previously noted in the original outcome study^37^ and include: (i) the DPAS, was not designed for a youth population; (ii) our self-report measures have not been validated for use with youth or CHR populations^63^; (iii) the study was possibly underpowered as we had planned 62 per arm at the final follow-up and more participants may have been needed to detect a difference; and (iv) the groups may have worked better as 18-week closed groups versus having rolling starts every 6 weeks to fit with the CBSST modules, a compromise between completely open and completely closed groups to avoid making CHR participants wait up to 18 weeks before starting a new group. Since there were three CBSST modules and participants who dropped out prior to 18 weeks would not necessarily have participated in the same modules. One limitation specific to this study is that negative symptoms were examined using the SOPS which is considered to be suboptimal, and a preferred scale may be the Negative Symptom Inventory-Psychosis Risk.^64–66^

Recommendations include further exploration into the impact of mediators on experiential negative symptoms. To avoid the problems of a closed 18-week group or a group with rolling starts every 6 weeks, another recommendation might be to test the effect of a group with approximately 12 sessions and more emphasis on cognitive skills to ensure that everyone receives the same sessions and possibly allow for improved attendance. Finally, it is not unusual in psychotherapy to offer individual therapy first so that the person can make the most gains from a subsequent group therapy approach. It may be that for CHR youth with more severe APS, a few sessions of individual therapy, possibly CBT might better prepare them for entering a CBSST group.

## Conclusion

Key findings from this study were that, except for APS, none of the proposed mediator variables were significant. However, at the end of treatment, more severe APS were found to mediate the impact of treatment on social and role functioning and negative symptoms. This may offer some insights for the development of targeted treatments in future psychosocial trials involving CHR individuals. It is possible that a shorter intervention with more emphasis on cognitive skills could be an advantage or offering brief individual therapy to better prepare participants for a group intervention.

## Supplemental Material

sj-docx-1-cpa-10.1177_07067437241295636 - Supplemental material for Cognitive-Behavioural Social Skills Training: Mediation of Treatment Outcomes in a Randomized Controlled Trial for Youth at Risk of Psychosis: L’entraînement aux compétences sociales cognitivo-comportementales : variables médiatrices des résultats thérapeutiques dans le cadre d’un essai clinique randomisé pour les jeunes présentant un risque de psychose Supplemental material, sj-docx-1-cpa-10.1177_07067437241295636 for Cognitive-Behavioural Social Skills Training: Mediation of Treatment Outcomes in a Randomized Controlled Trial for Youth at Risk of Psychosis: L’entraînement aux compétences sociales cognitivo-comportementales : variables médiatrices des résultats thérapeutiques dans le cadre d’un essai clinique randomisé pour les jeunes présentant un risque de psychose by Daniel J. Devoe, Lu Liu, Amy Braun, Kristin S. Cadenhead, Barbara A. Cornblatt, Eric Granholm and Jean Addington in The Canadian Journal of Psychiatry
