# Supplementary material for: Cognitive-Behavioural Social Skills Training: Mediation of Treatment Outcomes in a Randomized Controlled Trial for Youth at Risk of Psychosis: L’entraînement aux compétences sociales cognitivo-comportementales : variables médiatrices des résultats thérapeutiques dans le cadre d’un essai clinique randomisé pour les jeunes présentant un risque de psychose
Source: Can J Psychiatry. 2024 Nov 11;70(3):217–28. doi: 10.1177/07067437241295636 (PMC11562941; doi:10.1177/07067437241295636)
Supplement: sj-docx-1-cpa-10.1177_07067437241295636 - Supplemental material for Cognitive-Behavioural Social Skills Training: Mediation of Treatment Outcomes in a Randomized Controlled Trial for Youth at Risk of Psychosis: L’entraînement aux compétences sociales cognitivo-comportementales : variables médiatrice [file sj-docx-1-cpa-10.1177_07067437241295636.docx]

**SUPPLEMENTARY MATERIAL**

**Content of CBSST Sessions**

**Cognitive Skills Module**

Session 1: INTRODUCTION AND GOAL SETTING

- Agenda
- Agenda Setting
- Introductions
- Using Your Workbook
- Group Contract
- What is Cognitive Behavioral Social Skills Training (CBSST)?
- What is Unhelpful Thinking?
- Vulnerability-Stress Model
- Setting Goals
- At-Home Practice: Setting Goals

Session 2: THE THOUGHT-FEELING- BEHAVIOR LINK

- Agenda
- What Is the Thought-Feeling-Behavior Link?
- Thoughts Versus Feelings
- Summary
- At-Home Practice: Identifying Thoughts

Session 3: THE 3C’s - CATCH-IT, CHECK-IT, CHANGE-IT

- Agenda
- The 3C’s: Catch-It, Check-It, Change-It
- Catch-It: Recognizing Unhelpful Thoughts
- Automatic Thoughts
- Common Thoughts Checklist
- Catch-It: Using Feelings as a “Red Flag”
- Summary
- At-Home Practice: Catch The Thought!

Session 4: THE 3C’s - CHECK-IT

- Agenda
- Check-It
- Mistakes In Thinking

Session 5: THE 3C’s - CHANGE-IT

- Agenda
- Change-It
- Generating Alternatives
- Overcoming Unhelpful Thoughts
- At-Home Practice: Catch, Check, and Change The Thought!

Session 6: 3C’s PRACTICE

- Agenda
- Doing Experiments to Check-It
- Practice Using the 3C’s
- The 3C’s and Your Goals
- At-Home Practice: Catch, Check, and Change The Thought!

**Problem Solving Skills Module**

Session 1: INTRODUCTION AND GOAL SETTING

- Introductions and Group Rules
- Using Your Workbook
- Group Contract
- What is Cognitive Behavioral Social Skills Training (CBSST)?
- What is Unhelpful Thinking?
- Getting What You REALLY Want
- At-Home Practice: Getting What You Really Want

Session 2: SOLVING PROBLEMS EFFECTIVELY

- Review At-Home Practice
- Strategies for Problem Solving
- My Problem-Solving Strengths and Weaknesses

Session 3: INTRODUCTION TO PROBLEM-SOLVING SKILLS

- Review At-Home Practice
- Steps to Follow to Solve Problems: SCALE
- At-Home Practice: SCALE Problem-Solving Worksheet

Session 4: SPECIFY THE PROBLEM

- Review At-Home Practice
- How to Specify the Problem
- How Do Your Thoughts Relate To Your Problems?
- Goal Obstacles Are Problems to Solve
- At-Home Practice: SCALE Problem-Solving Worksheet

Session 5: CONSIDER ALL SOLUTIONS

- Review At-Home Practice
- Consider ALL Solutions First, Then Assess
- Cognitive Flexibility
- Assess the Best Solution
- At-Home Practice: SCALE Problem-Solving Worksheet

Session 6: SOLVING PROBLEMS RELATED TO GOALS

- Review At-Home Practice
- Steps to Follow to Solve Problems: SCALE
- Solving Problems Related to Your Goals
- Planning to Meet Goals and Deadlines
- At-Home Practice: SCALE Problem-Solving Worksheet

**Social Skills Module**

Session 1: INTRODUCTION AND GOAL SETTING

- Introductions
- Using Your Workbook
- Group Contract
- What is Cognitive Behavioral Social Skills Training (CBSST)?
- What is Unhelpful Thinking?
- Setting Goals
- At-Home Practice: Setting Goals

Session 2: COMMUNICATE EFFECTIVELY TO ACHIEVE YOUR GOALS

- Review At-Home Activities
- Why is it Important to Communicate Well with Others?
- Communication Behaviors
- Active Listening
- Practicing Communication Behaviors and Active Listening
- At-Home Practice: Active Listening

Session 3: FACE-TO-FACE CONVERSATIONAL SKILLS

- Review At-Home Activities
- Why Are Conversational Skills So Important
- Starting, Maintaining & Ending Conversations
- At-Home Practice: Starting, Maintaining & Ending Conversations

Session 4: THE POWER OF EMOTIONS

- Review At-Home Activities
- The Power of Emotions
- Ten Action Signals: “Red Flag Feelings”
- Taking Action
- At-Home Practice: Taking Action

Session 5: GETTING PEOPLE TO STOP DOING STUFF THAT BOTHERS YOU

- Review At-Home Activities
- “I” statements
- Expressing Negative Feelings
- At-Home Practice: Expressing Negative Feelings

Session 6: STICKING TO YOUR LIMITS

- Review At-Home Activities
- Boundaries
- Who’s Got Your Back?
- Sticking to Your Limits
- Remembering Your Rights
- Tip: Weigh the Pros and Cons
- At-Home Practice: Sticking to Your Limits

**MANUAL ADAPTATION**

Cognitive-behavioral social skills training (CBSST) was adapted for younger individuals at clinical high-risk (CHR) for psychosis. In their CBSST manual for individuals with schizophrenia, Granholm et al. (2016) recommends that participants complete eighteen sessions and then possibly re-enroll for a second time, meaning they will attend thirty-six sessions in total. Because cognitive deficits are not as severe among the CHR population, a second exposure was not deemed necessary. Thus, the ReGroup participants completed the three 6-week modules at once. Most of the content was maintained from the original version, although some sessions covered the material in more detail or less detail. Changes to make the material more youth focused included the types of activities and behavioral examples used. Rapidly evolving youth culture meant facilitators tried to let youth contribute in ways that allowed examples and solutions discussed during group sessions to be current. For instance, while older populations might talk about calling a friend on the phone, most youth do not call people anymore. Instead, they might interact through text, or even limit communication to “snapping” on Snapchat. While some TV shows might be popular with adults, youth were more likely to talk about their favorite YouTuber or influencer. Facilitators strove to create a familiar and relaxed atmosphere in group sessions to prevent it from feeling like school. Finally, role and social functioning emerged organically and were themes often discussed in the groups denoting the importance of school, work, and social relationships for these youth.

Some examples of adaptations made in each module include but are not limited to:

**Goal setting:**

- The goal setting is covered in the first session of each module. The adapted manual guided participants to focus on living, learning, working and socializing areas of life (not symptoms), that tend to affect young people (e.g., school, family, work, romantic relationships, and friends). It is emphasized that goals should be valuable to the participant themself and not represent goals their parents might have for them. A further difference in the facilitation of goal setting sessions is that facilitators focused on developmentally relevant issues and events such as increasing autonomy from the family and strategizing steps related to career aspirations and adjusting to post-secondary education.

**Cognitive skills module:**

- Due to the cognitive deficits experienced by individuals with chronic schizophrenia, certain techniques included in the original manual were removed for the CHR population. For example, goal setting covered in the cognitive skills module (Session 1: *Introduction and goal setting*) was explained to the chronic population using the 7-7-7 Goal Jackpot mnemonic. This was removed for the CHR groups, where goal setting was only described as short- and long-term goals.
- The CHR manual includes a more detailed background of CBSST, unhelpful thoughts, and unusual experiences.
- Examples of “catching unhelpful thoughts” (Session 3: *The 3 C’s Catch-it, Check-it, Change-it*) were adapted from the original manual. While the original manual included some focus on symptom related experiences such as voices, paranoia, or being afraid of meeting someone new, the adapted manual focused more on learning new skills, thoughts regarding the school environment, going to a party, or being assertive.

● Most of the cognitive skills were taught through games and exercises adapted to engage a younger population:

- “Jenga tower.” In this game, thoughts and feelings were written on wood blocks from the commercial Jenga game, and as they were pulled from the tower, the participant was asked to identify whether the block contained a thought or a feeling.
- “Catching mistakes in thinking.” In this game, a group member is tossed a crumpled piece of paper with an example thought for a specific mistake in thinking written on it (e.g., “I will never make a friend”) and are asked what mistake in thinking it is (e.g., fortune-telling).
- “Heads Up.” In this game, participants wore hats with a type of cognitive distortion (e.g., all or nothing thinking, fortune-telling, or emotional reasoning) written on it. Without knowing what their hat said, participants had to guess based on descriptive clues given to them by other group members.

**Social skills module:**

- Basic social skills were covered early in both versions of this module, although they were less pertinent for the CHR group. Considering this, some aspects were relaxed; for example, the instruction in the original manual to avoid staring directly at people was modified for the CHR population to advise them to maintain good eye contact.
- Many CHR participants had adequate basic social skills and could integrate instructions for appropriate communication into their role-play well. While the original manual stressed practicing role-play three times with feedback on each attempt, many CHR youth made the necessary changes by the second role-play. Facilitators would then increase the difficulty and use social skills role-play as an opportunity to practice addressing specific issues related to a participant’s goals. The participant could then apply the role-play experience to their “at-home practice” that week, as they worked towards achieving a goal. For example, if a participant were having an ongoing issue setting boundaries with a parent, they could role-play having a difficult conversation during a group with another group member standing in as the parent. Bolstered by that role-play, the participant could use the experience and skills from session 5 to attempt to initiate that conversation effectively and appropriately with their parents in real life.
- The original manual focused on effective communication, expression of pleasant and unpleasant feelings, making positive requests, and asking for help with their goals. While most of this was covered within the adapted manual, session content differed. The adapted manual did not include a session on how to ask for help with goals or on explicitly expressing positive emotions. Instead, it focused on skills such as starting, maintaining, and ending conversations, identifying, coping with, and communicating about a range of feelings, setting healthy boundaries, and identifying members of the individual’s support structure.

**Problem-solving module:**

- CHR participants are asked to engage more thoroughly with their problem-solving strategies. For example, after brainstorming a list of possible solutions, the group discussed whether solutions are “passive” or “active”, and the benefits and drawbacks of these divergent strategies. This session (*Session 2: Solving problems effectively*) was not covered in the original manual.
- Example of problems outlined in the manuals differ in the following ways:
- Medication use (remembering to take it, making decisions about what/when to take it) has a greater focus in the original manual than in the adapted version, as it is much less common in the CHR population to be taking psychotropic medication regularly.
- There is an increased focus on concerns related to socializing and peer/intimate relationships for the CHR population.
- Schoolwork and finding a first job (i.e., how to write a resume) are raised as possible issues for the CHR population.
- No mention of issues related to positive symptoms appears in the adapted CHR manual, whereas the original uses some examples (i.e., focus on perceptual abnormalities as a problem to address).
- There is an increased focus on difficulties achieving independence from parents among the CHR population.
- More time spent on more intricate problems among CHR youth.

**References**

Granholm, E., McQuaid, J., & Holden, J. (2016). *Cognitive Behavioral Social Skills Training for schizophrenia: A practical treatment guide*. Guilford Press.

| **Supplementary Table 1. Comparisons between participants with follow-up and retained in study and those who were not included in final analyses** | | | | | |
| --- | --- | --- | --- | --- | --- |
| **Variable** | | **Removed**  **n = 51** | **Retained**  **n = 152** | **Test Statistic** |  |
|  | | *Mean (SD)* | *Mean (SD)* | *t* | *P value* |
| Age (years) | | 17.31 (3.62) | 17.42 (4.06) | -0.18 | 0.859 |
| Years of education | | 10.51 (2.48) | 10.38 (2.65) | 0.30 | 0.762 |
| GF:S | | 5.84 (1.19) | 5.91 (1.29) | -0.32 | 0.753 |
| GF:R | | 5.04 (2.12) | 5.59 (2.35) | -1.49 | 0.139 |
| SOPS positive | | 11.31 (3.35) | 10.37 (4.21) | 1.45 | 0.147 |
| SOPS negative | | 13.33 (5.88) | 11.52 (6.34) | 1.80 | 0.073 |
| IQ | | 99.40 (14.45) | 103.50 (13.70) | -1.73 | 0.085 |
|  | | *Frequency (%)* | *Frequency (%)* | χ*^2^* | *P value* |
| Sex | |  |  |  |  |
|  | Male | 30 (58.8) | 69 (45.4) | 2.76 | 0.097 |
|  | Female | 21 (41.2) | 83 (54.6) |  |  |
| Race | |  |  |  |  |
|  | Caucasian | 34 (66.7) | 92 (60.5) | 0.75 | 0.686 |
|  | Black | 4 (7.8) | 17 (11.2) |  |  |
|  | Other^a^ | 13 (25.5) | 43 (28.3) |  |  |
| Marital status | |  |  |  |  |
|  | Single/never married | 48 (96) | 146 (96.1) | 1.61 | 0.447 |
|  | Married/common law | 2 (4.0) | 3 (2.0) |  |  |
| Living arrangement | |  |  |  |  |
|  | Living with family | 46 (90.2) | 134 (88.2) | 4.18 | 0.382 |
|  | Living with spouse/partner | 2 (3.9) | 8 (5.3) |  |  |
|  | Living on own | 0 (0.0) | 3 (2.0) |  |  |
|  | Living with others^b^ | 2 (3.9) | 7 (4.6) |  |  |
| Education Completed | |  |  |  |  |
|  | Grade school | 35 (68.6) | 101 (66.5) | 2.1 | 0.717 |
|  | High school | 13 (25.5) | 41 (27.0) |  |  |
|  | College | 2 (3.9) | 9 (5.9) |  |  |
|  | Technical school | 1 (2.0) | 1 (0.7) |  |  |
| Current employment | |  |  |  |  |
|  | Working full time | 3 (5.9) | 6 (4.0) | 0.73 | 0.867 |
|  | Working part-time | 7 (13.7) | 24 (15.8) |  |  |
|  | Worked in past year | 9 (17.7) | 32 (21.1) |  |  |
|  | Not worked in past year | 32 (62.8) | 90 (59.2) |  |  |
| *^a^ Includes First Nations, East Asian, Southeast Asian, South Asian, West/Central Asian, and Middle Eastern, Native Hawaiian or Pacific Islander, Interracial*  *^b^ Includes living with friends (excluding spouse/partners), in a boarding/group home, or academic residence.*  *GF:S, Global Functioning: Social; GF:R, Global Functioning: Role; SOPS, Scale of Psychosis-Risk Symptoms.* | | | | | |

| **Supplementary Table 2. Differences in clinical variables within and between groups** | | | | | | | |
| --- | --- | --- | --- | --- | --- | --- | --- |
| Variables | | CBSST (n=70) | | | ST (n=82) | | |
|  | | Baseline  (n=70) | End of Treatment  (n=66) | 12 months  (n=57) | Baseline  (n=82) | End of Treatment  (n=78) | 12 months  (n=66) |
|  | | *Mean (SE)* | *Mean (SE)* | *Mean (SE)* | *Mean (SE)* | *Mean (SE)* | *Mean (SE)* |
| GF:S | | 5.89 (0.16) | 6.42 (0.16)^a**^ | 6.52 (0.19)^a**^ | 5.93 (0.14) | 6.21 (0.15) | 6.34 (0.17) |
| GF:R | | 5.97 (0.28) | 6.62 (0.26) | 6.19 (0.28)^b**^ | 5.27 (0.26) | 5.80 (0.24) | 6.07 (0.26)^a*^ |
|  | |  |  |  |  |  |  |
| DPAS | | 55.43 (2.32) | 49.89 (2.21)^a*^ | 49.20 (2.45) | 55.27 (2.16) | 53.86 (2.07) | 53.05 (2.28) |
|  |  |  |  |  |  |  |  |
| SOPS + | | 9.89 (0.50) | 6.83 (0.50)^a***^ | 5.91 (0.51)^a***^ | 10.78 (0.46) | 8.16 (0.46)^a***^ | 6.86 (0.47)^a***b**^ |
| SOPS - | | 11.11 (0.76) | 8.58 (0.74)^a**^ | 8.14 (0.80)^a***^ | 11.87 (0.70) | 9.93 (0.68)^a*^ | 9.41 (0.74)^a**^ |
| CDSS | | 6.16 (0.61) | 4.15 (0.50)^a*^ | 3.35 (0.44)^a***^ | 6.02 (0.56) | 4.30 (0.46)^a*^ | 3.25 (0.41)^a***^ |
| SAS | | 40.49 (1.49) | 36.70 (1.47)^a*^ | 35.60 (1.47)^a**^ | 40.28 (1.39) | 36.75 (1.37)^a*^ | 36.17 (1.36)^a*^ |
| SIAS | | 35.37 (2.34) | 30.09 (2.35)^a*^ | 28.71 (2.23)^a**^ | 35.23 (2.18) | 32.48 (2.20) | 32.43 (2.06) |
|  | |  |  |  |  |  |  |
| SSES | | 53.04 (2.81) | 59.27 (2.86)^a*^ | 57.85 (2.93) | 51.71 (2.59) | 56.56 (2.69) | 56.03 (2.71) |
| ABS | | 7.35 (0.41) | 6.94 (0.42) | 6.92 (0.45) | 7.52 (0.38) | 7.05 (0.39) | 7.25 (0.41) |
| BCSS other | | 7.87 (0.74) | 6.93 (0.72) | 5.27 (0.76)^a*^ | 6.72 (0.69) | 6.21 (0.68) | 7.00 (0.69) |
| BCSS self | | 6.62 (0.80) | 5.91 (0.79) | 4.36 (0.70) | 7.88 (0.74) | 6.68 (0.74) | 5.73 (0.64)^a*^ |
|  | |  |  |  |  |  |  |
| EDF40 | | 22.89 (0.66) | 22.48 (0.70) | 22.67 (0.75) | 24.03 (0.61) | 23.93 (0.65) | 24.03 (0.70) |
| ER40 | | 33.17 (0.45) | 33.85 (0.43) | 34.65 (0.42)^a*^ | 32.69 (0.42) | 33.12 (0.40) | 34.17 (0.39)^a**^ |
| TASIT | | 51.19 (0.75) | 52.58 (0.80) | 53.71 (0.80)^a***^ | 51.66 (0.70) | 53.37 (0.74)^a*^ | 53.46 (0.70) ^a*^ |
| Abbreviations: Mean represents the least squares means estimated by the generalized linear model, SE represents the standard error of the mean; GF:S, Global Functioning: Social; GF:R, Global Functioning: Role; SOPS +, Scale of Psychosis-Risk Symptoms Positive symptoms; SOPS -, Scale of Psychosis-Risk Symptoms Negative symptoms; CDSS, Calgary Depression Scale for Schizophrenia; SAS, Social Anxiety Scale; SIAS, Social Interaction Anxiety Scale; DPAS, Defeatist Performance Beliefs Scale; SSES, Social Self Efficacy Scale; ABS, Asocial Beliefs Scale; BCSS, Brief Core Schema Scale; EDF40, Emotion Differentiation; ERF40, Emotion Recognition; TASIT, The Awareness of Social Inference Test. Note: SOPS Negative Symptoms (SOPS -) includes total scores of N1-N6.  Significance: a= significantly different from baseline; b= significantly different from end of treatment. *p≤0.05, **p≤0.01, ***p≤0.001 | | | | | | | |
